# Automatic Localization of Five Relevant Dermoscopic Structures Based on YOLOv8 for Diagnosis Improvement

**DOI:** 10.3390/jimaging9070148

**Published:** 2023-07-21

**Authors:** Esther Chabi Adjobo, Amadou Tidjani Sanda Mahama, Pierre Gouton, Joël Tossa

**Affiliations:** 1 Imagerie et Vision Artificielle (ImVia), University of Bourgogne Franche-Comté, 21078 Dijon, France; sandatidjani@gmail.com (A.T.S.M.); pierre.gouton@u-bourgogne.fr (P.G.); 2 Institut de Mathématiques et de Sciences Physiques (IMSP), University of Abomey-Calavi, Abomey-Calavi BP 2549, Benin; joel.tossa@gmail.com

**Keywords:** features detection, single-shot object detection algorithm, dermoscopic images

## Abstract

The automatic detection of dermoscopic features is a task that provides the specialists with an image with indications about the different patterns present in it. This information can help them fully understand the image and improve their decisions. However, the automatic analysis of dermoscopic features can be a difficult task because of their small size. Some work was performed in this area, but the results can be improved. The objective of this work is to improve the precision of the automatic detection of dermoscopic features. To achieve this goal, an algorithm named yolo-dermoscopic-features is proposed. The algorithm consists of four points: (i) generate annotations in the JSON format for supervised learning of the model; (ii) propose a model based on the latest version of Yolo; (iii) pre-train the model for the segmentation of skin lesions; (iv) train five models for the five dermoscopic features. The experiments are performed on the ISIC 2018 task2 dataset. After training, the model is evaluated and compared to the performance of two methods. The proposed method allows us to reach average performances of 0.9758, 0.954, 0.9724, 0.938, and 0.9692, respectively, for the Dice similarity coefficient, Jaccard similarity coefficient, precision, recall, and average precision. Furthermore, comparing to other methods, the proposed method reaches a better Jaccard similarity coefficient of 0.954 and, thus, presents the best similarity with the annotations made by specialists. This method can also be used to automatically annotate images and, therefore, can be a solution to the lack of features annotation in the dataset.

## 1. Introduction

In skin imaging, a common modality for visualizing tissue structure is dermoscopy. Dermoscopy is a non-invasive method that allows for the observation of more information about the skin structure, and this observation can be saved as an image. A dermoscopic image is characterized by the presence of dermoscopic structures (patterns) used by specialists to make diagnoses. Some dermoscopic structures present in malignant lesions are the pigment network, dots, globules, and streaks.

**Figure 1:**
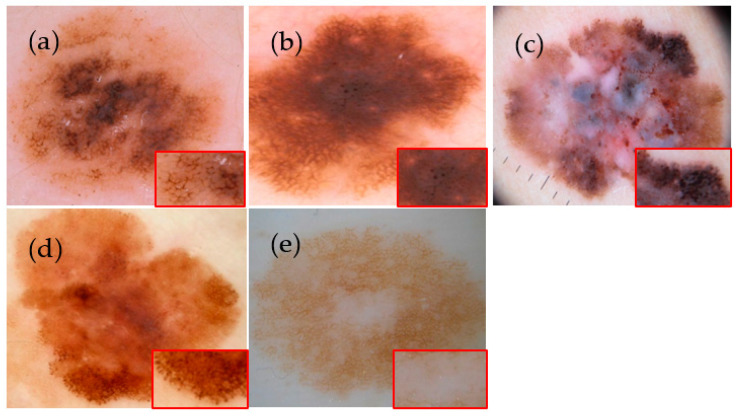
(**a**) Atypical pigment network on a dermoscopic image of nevus, (**b**) Irregular dots on a dermoscopic image of nevus, (**c**) Irregular globules on a dermoscopic image of melanoma, (**d**) Streaks on a dermoscopic image of melanoma, (**e**) Area without structure on a dermoscopic image of nevus [3,4,5].

The pigment network corresponds to the visualization of melanin located in the keratinocytes and in the melanocytes along the dermal–epidermal junction. It forms a honeycomb pattern with meshes (pigmented lines) and intermeshes (hypopigmented holes) [1]. The pattern formed can be regular (typical) or irregular (atypical). Dots [2] are small, round, well-circumscribed structures (less than 0.1 mm). These dots are said to be regular if they are grouped in the center of the lesion or located on the lines of the pigmentary network. Globules [2] are round to oval well-demarcated structures that are larger than 0.1 mm. When dots and globules are atypical (differences in size, shape, color, or distribution), the diagnosis of melanoma is preferred. Streaks [2] are linear pigmented projections that localize at the edge of lesions and include radial streaks (linear streaks) and pseudopodia (bulbous projections). Some lesions may also have areas without structures. Lesions with only a structureless pattern are difficult to diagnose by dermoscopy. This work is particularly interested in lesions or parts of lesions that have dermoscopic structures. Figure 1 shows some examples of dermoscopic structures.

The difficulty in the automatic detection of dermoscopic features is on several levels. First, dermoscopic features are generally small. For example, according to [2], dots are oval and round structures smaller than 0.1 mm and globules are oval structures larger than 0.1 mm. Second, dermoscopic structures are complex and their analysis requires some expertise [6]. In addition, there is a lack in annotated images. To train automatic detection algorithms, it is necessary to have large databases with labels. Finally, the existing annotations reveal that some features are more present than others.

The objective of this paper is to propose a high-performance method that estimates the location of five relevant dermoscopic features: globules, milia-like cysts, pigment networks, negative networks, and streaks. This work will help specialists to gain a complete understanding of the image and improve their decisions.

The contributions of this work are: (i) the generation of annotated images in the JSON format from annotations made by experts and provided in the ISIC challenge 2018 task 2 databases. This format is required for state-of-the-art detection algorithms. The resulting annotation will be made freely available for further research. (ii) This paper proposes a framework for the automatic detection of five dermoscopic features. (iii) This paper contains a review of the literature on the subject.

In the remainder of this paper, Section 2 reviews methods for detecting dermoscopic features and presents the proposed method. Next, Section 3 reports the experimental results, followed by Section 4, which discusses the results. Finally, there are a conclusion and perspectives.

## 2. Materials and Methods

### 2.1. Dermoscopic Structures Localization Methods

**Figure 2:**
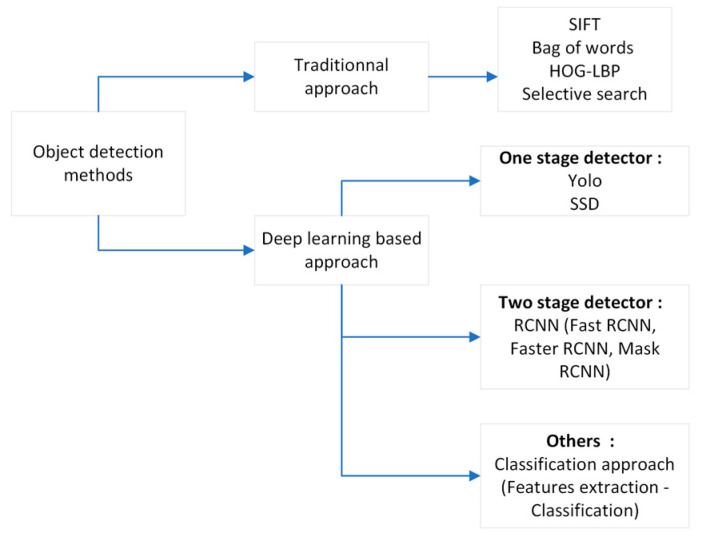
Classification of object detection methods.

In the last decade, several works and progress have been made on the localization of dermoscopic structures. Recent advances in these algorithms were made possible by the advent of deep learning methodologies. However, before deep learning was used, methods such as scale-invariant feature transform (SIFT) [7] and gradient histogram (HOG) [8] and support vector machine (SVM) [9] classifiers were widely used. Therefore, there are two categories of feature detection methods: traditional methods based on manual feature extraction and methods based on deep learning. Figure 2 lists these different families of feature detection methods. This section presents the recent literature on automatic localization in dermoscopic images.

#### 2.1.1. Traditional Object Localization Approach

Barata et al. in [10] proposed a method that detects a pigment network mask in two steps. The first step is to enhance the transitions between the dark lines and the lighter “holes” in the lesion image (to better detect the pigment network lines) using directional filters. Then, an algorithm based on the eight-connections criterion is applied to detect the large linked structures that represent the pigment network area. Pathan et al. in [11] applied a Gabor filter on the green channel of the lesion image to generate the pigment network mask. Garcia-Arroyo et al. in [12] proposed a pigment network recognition method that first uses a fuzzy classification model to classify each image pixel into three categories: “net”, “hole”, and “other”. Then, a set of color and texture features are extracted to discriminate between the absence and presence of the pigment network. Benam et al. in [13] introduced a system to locate and retrieve images with a similar pigment network with a query image. The algorithm first finds key points in the region using a blob detection approach, the same as in SURF. Then, using luminance and color components, it extracts 128 features as descriptors from each key point. Finally, the algorithm matched key points in the query image with similar key points appearing in images of the database. Delibasis et al. in [14] proposed a methodology based on Hessian multiresolution image features to automatically detect streaks in dermoscopy images. The detected features are used for the classification of lesions into malignant or non-malignant.

#### 2.1.2. Deep Learning Object Localization Approach

Jahanifar et al. in [15] proposed a five attribute segmentation algorithm based on encoder–decoder architecture to segment each dermoscopic attribute. The encoder uses a variety of pre-trained networks, and the decoder generates the prediction map by combining multi-scale information using a pyramid pooling approach. Sorokin et al. in [16] proposed a lesion attribute detection method based on the mask R-CNN model. The model was pre-trained on the COCO database [17] and detected five dermoscopic attributes. Li et al. in [18] tackled the task of dermoscopic feature extraction by proposing a patch-based CNN named the lesion feature network (LFN). The LFN framework first subdivided the image into 996 superpixel areas. Then, each superpixel area was classified into one of five categories: four types of dermoscopic features (pigment network, negative network, striae, and milia-like cysts) and background. Kawahara et al. in [19] implemented a convolutional-neural-network-based method to detect the presence of four dermoscopic criteria (pigment network, negative network, milia-like cysts, streaks) in a dermoscopic image. The CNN architecture uses interpolated maps and concatenated feature maps from the intermediate layers of the network. Their approach in 2017 won first place in the ISIC-ISBI Part 2 challenge. Bissoto et al. in [20] approached the lesion attribute detection task with a finely tuned Inception-v4 network pre-trained on ImageNet [21]. Each image was sliced into patches of dimensions 128 × 128 or 299 × 299, and each patch was submitted to the model for classification into six classes: absent, pigment network, negative network, milia-like cysts, streaks, and globules. Z. Chen et al. in [22] proposed a multi-task U-Net model to automatically detect dermoscopic attributes. The characteristic of the proposed model is that the U-Net encoding path was replaced by a pre-trained VGG16 model. Instead of using the pre-trained model with databases such as ImageNet or COCO, which are very different from medical datasets, Nguyen et al. in [23] proposed task agnostic transfer learning (TATL) to detect the five attributes of skin lesions. The proposed method is inspired by the behavior of dermatologists and consists of three networks. The first network segments the lesion regions in the image. Then, the second network identifies any abnormal positions in the segmented image. The last network is trained to detect only one attribute.

All these proposed methods based on deep learning have approached the dermoscopic feature localization as a classification task [20] or a segmentation task [15], working either on the whole image [16,18] or on the segmented lesion [19,23], or on superpixels [18,19,20]. Pre-processing methods were applied by some authors [16,18] to enlarge the lesion area for feature detection and to remove artifacts present on some images. Furthermore, at the end of the proposed frameworks, other authors [15,20] have applied post-processing to restrict the attribute prediction maps to the lesion area and to mitigate false positives that usually occur in abundant attributes.

Given the imbalance observed in the annotation of dermoscopic features in the two databases, other authors have proposed approaches to correct this. Kawahara et al. in [19] address the problem by minimizing a Sorensen–Dice coefficient, computed over the entire mini-lot for each label. Bissoto et al. [20] attempted to address the imbalance in the dataset; they formed balanced batches of data that were used to train each model. Li et al. in [18] also tried to solve the problem by assigning weights to the different classes (attributes) when calculating the softmax loss in order to give more attention to classes with fewer samples. A common feature of all these references is the database. Most of the works cited exploit the databases provided by the ISIC competition (ISIC 2017 [3] or ISIC 2018 [5,24]). These datasets have the potential to advance research on the topic. However, not all dermoscopic structures are annotated in these databases and the number of examples for most annotated structures is small. Furthermore, for dermatologists, providing local annotations is a cumbersome, tedious, and time-consuming task [25].

Given these challenges, this paper proposes a method to improve the automatic localization of dermoscopic features and to auto-generate dermoscopic feature annotations.

### 2.2. Dataset

The used dataset is from the ISIC 2018 challenge and contains 2594 skin lesion images and 12,970 masks corresponding to the annotated dermoscopic features (five for each image). The masks were obtained from expert annotations, with a cross-validation from multiple evaluators. The five annotated dermoscopic features are globules, pigment network, negative network, streaks, and milia-like cysts. The resolution of dermoscopic images in the dataset vary between 640 × 480 and 1944 × 2592. On the dataset, 603 images show globules, 1523 show a pigment network, 191 show a negative network, 100 show streaks, and 682 show milia-like cyst features. No pre-processing is applied to the images in the database because, on the one hand, they rarely contain hairs that would interfere with visual and automatic analysis. Second, to avoid removing the desired features. Indeed, the pigment network and the negative network have a brown line pattern like hairs and can be assimilated to them and removed during pre-processing.

**Figure 3:**
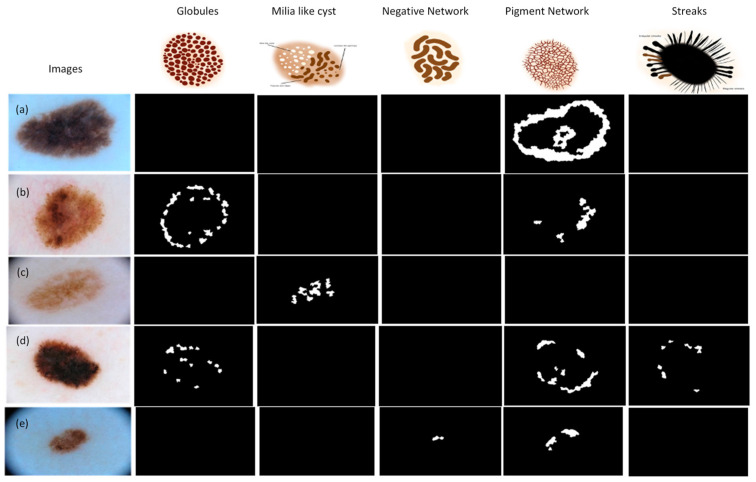
Features structures and dataset images [3,4,5]. (**a**) Dermoscopic image of melanocytic nevi, (**b**) Dermoscopic image of melanoma, (**c**) Dermoscopic image of nevus, (**d**) Dermoscopic image of melanoma, (**e**) Dermoscopic image of nevus.

Figure 3 presents the feature structures, some dermoscopic images of the dataset, and the corresponding ground truth response masks (dark images represent the absence of the feature in the image).

### 2.3. Proposed Approach

**Figure 4:**
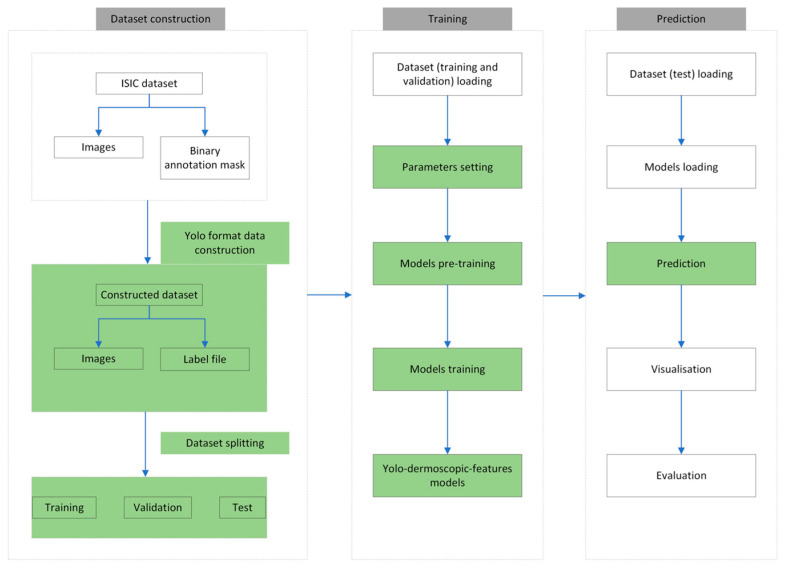
Pipeline of the YDL approach. The green modules represent parts on which modifications were made.

The pipeline of the proposed approach, named Yolo-Dermoscopic-Feature (YDL), like all deep learning approaches, consists of three parts: dataset construction (organization), training, and prediction. Figure 4 presents the different steps of the Yolo-dermoscopic-feature algorithm. The green modules represent the parts on which modifications were made.

#### 2.3.1. Dataset Construction

The dataset construction part consists of generating the labels files from binary masks. The binary masks were obtained from expert annotations. The file format used in this work is the JavaScript Object Notation (JSON). JSON is a lightweight data exchange format based on a subset of the JavaScript programming language standard ECMA-262 [26]. Algorithm 1 resumes the different steps to obtain the file.
**Algorithm 1:** Generate the label file from the binary images.Input: binary mask and the number of binary images Ni Output: file Initialization of the number of contours in an image Nc to 0 For each image i from 1 to Ni    Nc = Detection of the number of contours in the mask    file = Creation and initialization of the output file    For each image j from 1 to Nc      coordinates = Determination of the contour coordinates      file = Saving the coordinates on the file end Closing the file end

**Figure 5:**
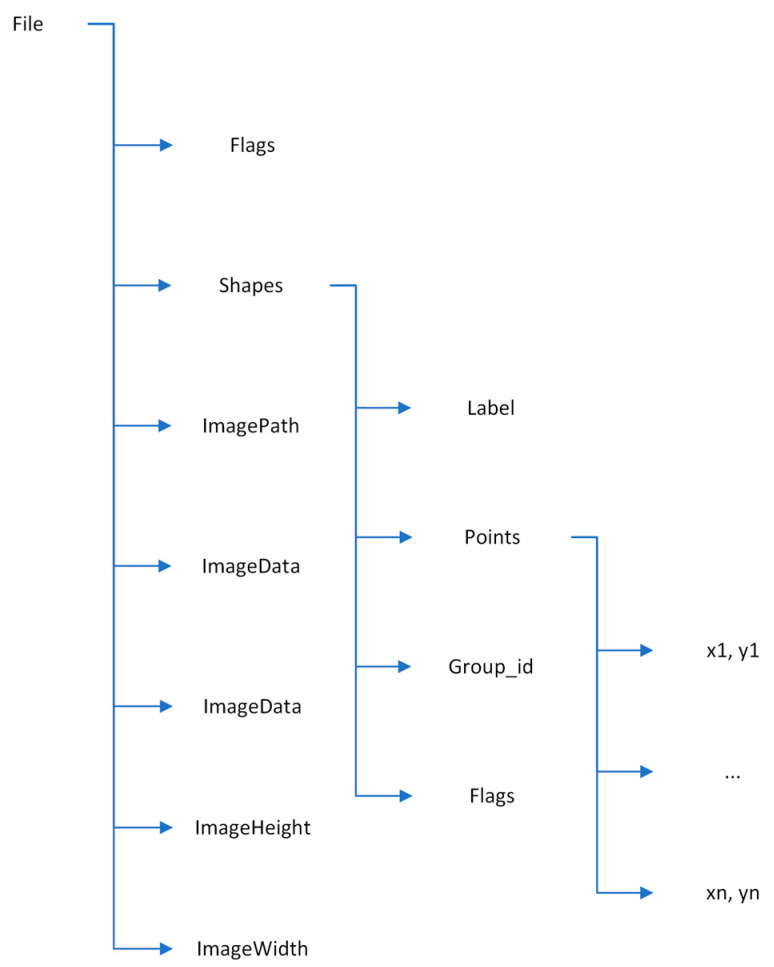
Hierarchical structure of the generated JSON file.

The generated file contains, among other things, information on the types of dermoscopic features present in the image (label), the coordinates of the points that delimit the dermoscopic feature (points), and the dimensions of the image (imageHeight and imageWidth). Figure 5 shows the structure of the generated file.

#### 2.3.2. Yolo Algorithm

**Figure 6:**
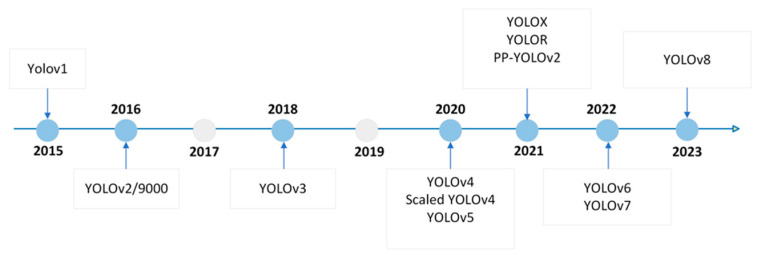
Timeline of different versions of Yolo.

You Only Look Once (Yolo) is an object detection and image segmentation model developed at the University of Washington by Joseph Redmon and Ali Farhadi. The first version of Yolo [24] was released in 2015, and since then, updates have been made to the architecture and cost functions to improve its accuracy and performance. Figure 6 shows the timeline of the different versions of Yolo.

**Figure 7:**
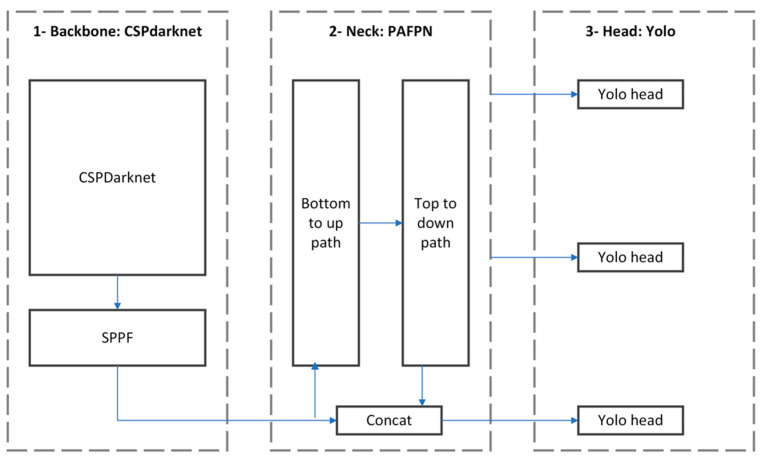
Yolo Architecture.

This algorithm, as presented in Figure 7, incorporates three parts, a backbone (or dorsal spine) network, a neck (or detector neck) network, and a detector head network. The backbone is a feature extractor module based on deep learning architecture. All backbone models are basically classification models similar to visual geometry group VGG [27], residual transformation block (ResNet) [28], dense convolutional network (DenseNet) [29], and cross stage partial network approach to Darknet (CSPDarknet) [30]. The neck module aggregate feature maps from different resolutions of the backbone fuses the features of different resolutions. Examples of the neck algorithm are the feature pyramid network (FPN) [31] and the path aggregation network (PAN) [32]. Finally, the head modules perform the object detection. Multiple heads are used to perform the prediction in different resolutions. Some of the head detectors are region proposal network (RPN) [33], Yolo [34].

In Yolov8, the CSPDarknet, PAFPN, and YoloHead networks are used as the core network, neck network, and head network, respectively. Yolov8 is the latest version of the Yolo detection models and is written and maintained by Jocher Glenn and other contributors from the company Ultralitycs [35]. Yolov8 is anchor-free and uses mosaic augmentation during training. The anchor-free approach simplifies the model by removing the anchor boxes. It allows the model to handle objects of different scales and aspect ratios without relying on prior information. Mosaic data augmentation involves combining multiple images into a single mosaic image and using it as input during training. Mosaic augmentation helps Yolov8 generalize better by forcing the model to learn from a more varied and challenging set of training samples. Yolov8 uses varifocal loss (VFL) as classification loss and distribution focal loss (DFL) and complete intersection over union loss (CIoU) as bounding boxes loss. These improvements make learning and inference much faster and improve the accuracy of the model. These are the reasons that motivated the choice of this algorithm.

In this work, instead of directly using a pre-trained ImageNet back-end, the model is first trained to segment the lesion area in the dermoscopic images. Then, the pre-trained model is used for dermoscopic feature localization. Furthermore, to correct the observed imbalance in features, five models are trained for the five dermoscopic features (globules, pigment network, negative network, milia-like cysts, and streaks). That means there is one model per feature.

## 3. Results

### 3.1. Experiments Setup and Evaluation Metrics

The experiments are performed on a machine with GPU NVIDIA A100 Tensor Core and 40GB RAM, and 70% of the dataset is used for training and 20% for validation and 10% for testing. The number of epochs is set to 100.

Three different losses are used to display model performance. These are: box loss (box_loss), segmentation loss (seg_loss), and classification loss (cls_loss). The box loss indicates how well the model can locate the center of an object and how well the predicted bounding box covers an object. Segmentation and classification losses indicate the ability of the model to, respectively, segment and predict the correct class of a given object.

Evaluation measures such as Dice similarity coefficient (DSC) [33], Jaccard similarity coefficient (JSC) [36], precision [25,37], recall [25,37], and average precision (mAP) [38] are used to evaluate the performance of detection with bounding boxes, segmentation, and feature classification. The average precision compares the ground truth bounding box to the detected box and returns a score. A high score means that the model is accurate in its detections. DSC and JSC are statistical measures used to evaluate the similarity of two samples. DSC, JSC, precision, recall, and average precision are, respectively, defined by Equations (1)–(5):

(1) $$DSC=\frac{2TP}{2TP+FP+FN}$$

(2) $$JSC=\frac{TP}{TP+FP+FN}$$

(3) $$Precision=\frac{TP}{TP+FP}$$

(4) $$Recall=\frac{TP}{TP+FN}$$

(5) $$Average\;precision=\frac{Precision+Recall}{2}$$

where TP are the true positives, FP the false positives, and FN the false negatives.

Finally, a comparative study is conducted to measure the performance of the proposed framework against two state-of-the-art methods.

### 3.2. Experimental Results

This section presents the experimental results obtained for each of the five dermoscopic features during the training and prediction phase.

#### 3.2.1. Training

**Figure 8:**
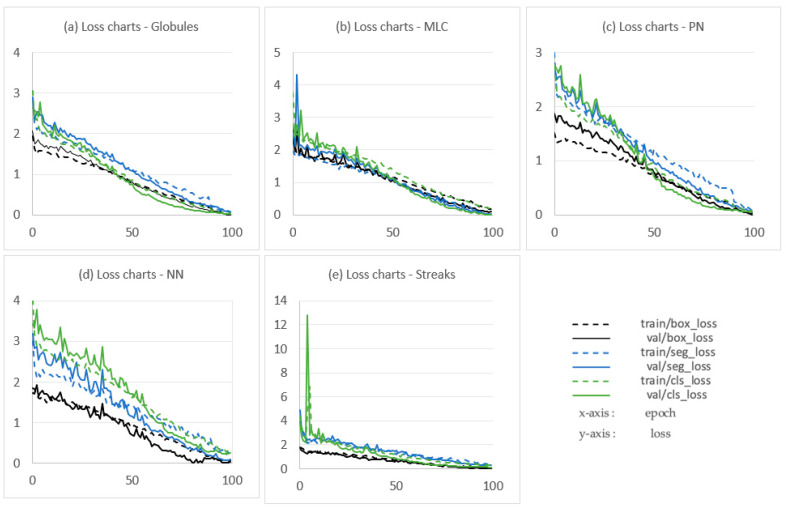
Loss charts of models during training and validation. (**a**), (**b**), (**c**), (**d**), and (**e**), respectively, represent charts of the globule localization model, the milia-like cyst (MLC) localization model, the pigment network (PN) localization model, the negative network (NN) localization model and the streak localization model. The epoch number is on the *x*-axis, and loss is on the *y*-axis.

The training or learning step consists of updating the parameters of a neural network to obtain the combination of parameters allowing the best possible prediction [39]. Figure 8 displays the loss charts of models during training and validation.

The loss functions all converge to zero from epoch 80 onwards. Furthermore, there is similarity between the pairs of training and validation curves. This means that the model generalizes well and fits the observed data on the training set, as well as the unobserved data on the validation set [40]. To avoid slowing down the learning speed, i.e., when the accuracy of the models stops improving after a certain time or even degrades due to noise learning (early stopping [40]), the number of epochs is limited to 100.

**Figure 9:**
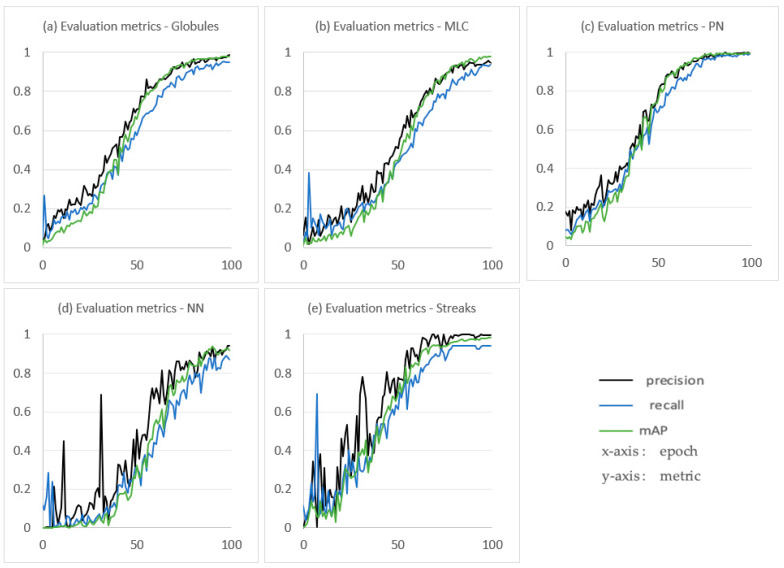
Performance measures of the models on training step. (**a**), (**b**), (**c**), (**d**) and (**e**), respectively, represent the performance metrics of the globule localization model, the milia-like cyst (MLC) localization model, the pigment network (PN) localization model, the negative network (NN) localization model, and the streak localization model. The epoch number is on the *x*-axis and the metric value (precision, recall, or mAP) is on the *y*-axis.

Figure 9 shows the performance measures during the training. The models start to improve in terms of precision, recall, and average precision after approximately 50 epochs. A stabilization is observed after approximately 80 epochs, and the measures converge to 1, which is the higher performance value. These curves allow us to observe that the learning process is progressive and that the model really improves at each epoch. The convergence of all the models towards 1 is consistent with the convergence of the loss curves (see Figure 8) towards 0 and motivates the choice to limit the number of epochs to 100 to avoid overfitting [25,40].

#### 3.2.2. Prediction

**Figure 10:**
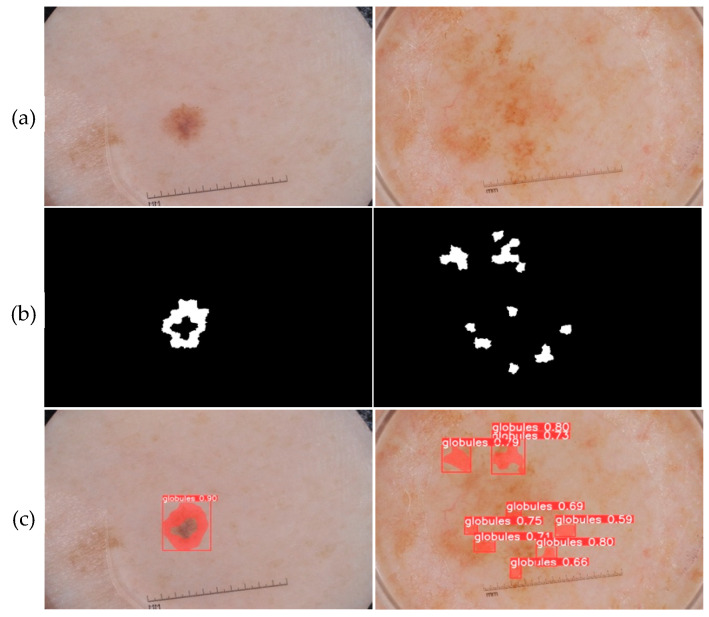
(**a**) Dermoscopic images. (**b**) Experts’ annotation masks of globules. (**c**) Predictions of globule positions.

**Figure 11:**
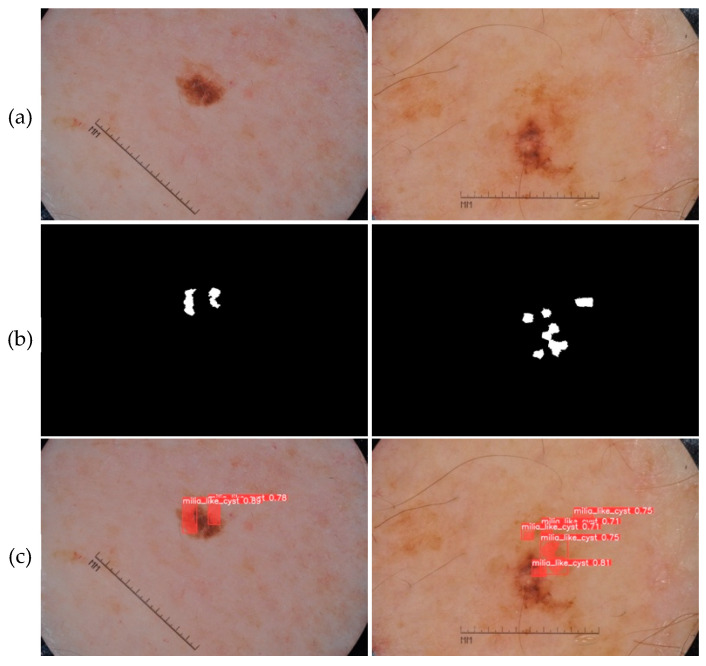
(**a**) Dermoscopic images. (**b**) Experts’ annotation masks of milia-like cyst (MLC). (**c**) Predictions of milia-like cyst (MLC) positions.

**Figure 12:**
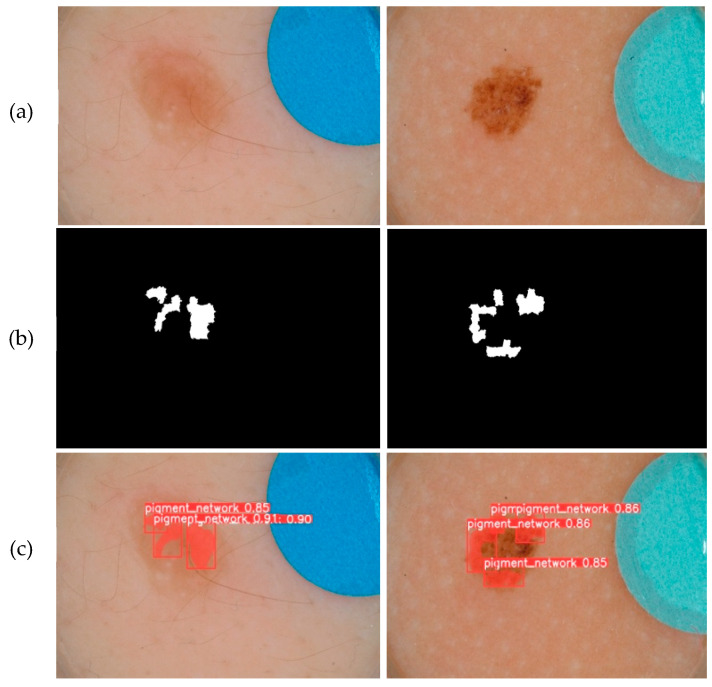
(**a**) Dermoscopic images. (**b**) Experts’ annotation masks of the pigment network (PN). (**c**) Predictions of pigment network (PN) positions.

**Figure 13:**
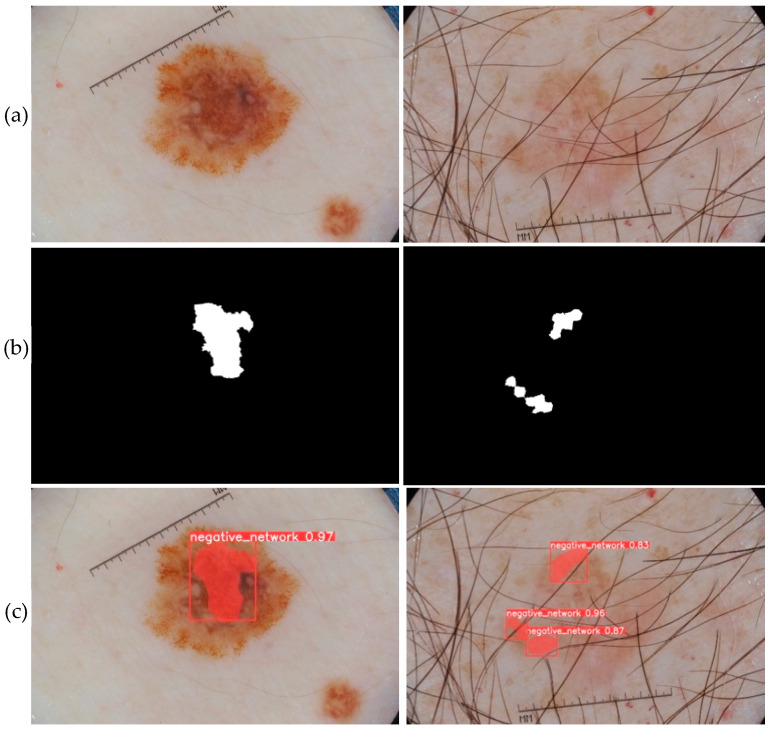
(**a**) Dermoscopic images. (**b**) Experts’ annotation masks of the negative network (NN). (**c**) Predictions of negative network (NN) positions.

**Figure 14:**
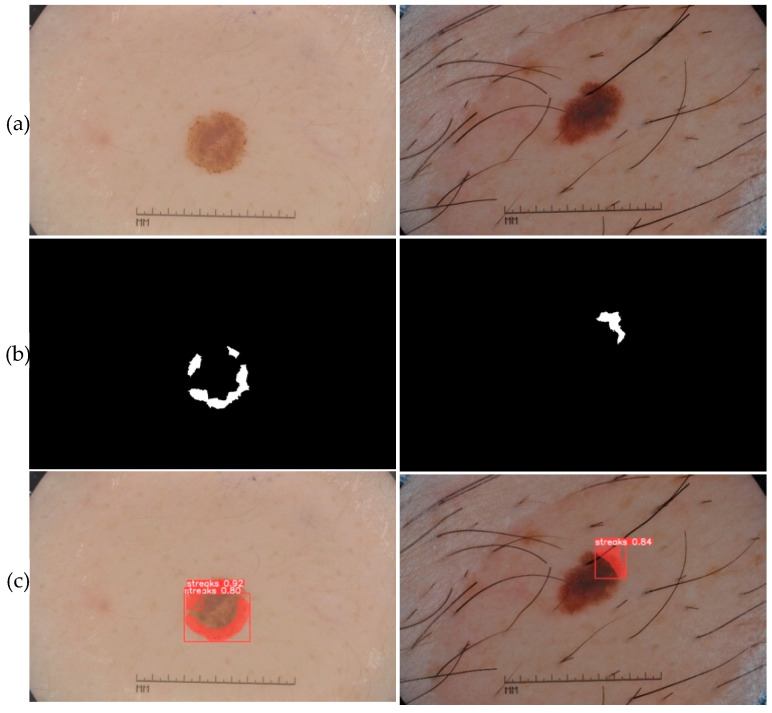
(**a**) Dermoscopic images. (**b**) Experts’ annotation masks of streaks. (**c**) Predictions of streaks positions.

The prediction phase follows the learning phase. The predictions were made on new images that were not used to train the models. The five trained models are first loaded. Then, based on the test set, each model makes predictions. Figure 10, Figure 11, Figure 12, Figure 13 and Figure 14 each consist of three lines. The first line shows example dermoscopic images, followed by the second line, which shows three binary masks corresponding to the three images in the first row. Finally, the last line shows the prediction results of the five features, respectively. For each prediction, the model return detected bounding boxes, the segmentation mask, and the corresponding predicted class and confidence scores. Moreover, the YDL algorithm allows for the generation of an annotation file based on the predictions. This label can then be used to annotate unannotated images and, thus, increase the number of annotated images.

##### Visual Evaluation

Looking at the prediction visually, we notice some strengths of the YDL algorithm:

- The segmentation masks for the predictions made (Figure 10c, Figure 11c, Figure 12c, Figure 13c and Figure 14c) show great similarities to the expert annotations (Figure 10b, Figure 11b, Figure 12b, Figure 13b and Figure 14b).
- The model detects well the presence of the dermoscopic structures in different regions.
- In some images, the features are located right on the edges. In these cases, the algorithm accurately detects the affected areas while delineating the central parts that do not have the feature.
- The method allows us to make predictions on images of small and large diameters.

##### Metrics Evaluation

Table 1 shows the performance of the models on the test set. The statistics presented correspond to the average obtained on all images on the test set. The calculated values for each of the metrics, DSJ, JSC, precision, recall, and average precision, converge to the highest performance score 1. The same is true for dermoscopic characteristics that are poorly represented in the database (negative network and streaks).

Based on the results obtained using the metrics, we can objectively conclude that the predictions made by the models have strong similarities with the annotations made by the experts. However, even if the results are interesting for all features, we notice a better performance for the dermoscopic structures that are well represented in the database.

##### Comparative Study

Finally, we compare the YDL algorithm results with two other state-of-the-art methods. The first method was proposed by Kawahara et al. [19] for detecting the presence of four dermoscopic criteria (pigmentary network, negative network, milia-like cysts, and streaks) in a dermoscopic image. Their approach in 2017 won first place in the ISIC-ISBI Part 2 challenge. The second method was proposed by Li et al. [18] for classifying four types of dermoscopic features (pigmentary network, negative network, streaks, and milia-like cysts) and the background.

Table 2 presents a comparison of the different dermoscopic feature extraction methods. We notice that all the methods are based on deep learning, use ISIC databases, and can detect several features. The YDL method is the one that extracts the most features (five features against four for the others). We make an estimation of the complexity of the three compared methods based on the feature extraction approach. Kawahara et al. [19] proposed a global approach for feature extraction similar to the proposed YDL method. Nevertheless, to improve the diagnostic accuracy, we chose to train multiple models, which increases the number of parameters to be trained and, consequently, the learning and prediction time. Li et al. in [18] chose a local approach for feature extraction, which means that they first subdivided the image into superpixel areas and then applied their method to each superpixel. This approach makes the algorithm more cumbersome and increases the learning time. Finally, we believe that the fastest and least complex method is that of Kawahara.

Table 3 presents the results of the comparisons. For JSC and the recall metrics, the proposed method shows interesting results. This result confirms the performance observed during the training and prediction phase.

## 4. Discussion

The results of the visual evaluation (Figure 10, Figure 11, Figure 12, Figure 13 and Figure 14), the metrics evaluation (Table 1), and the comparative study (Table 2 and Table 3) show interesting predictions with YDL compared to other methods. Averaging the results for each of the dermoscopic features, we obtain 0.9758 for DSC, 0.954 for JSC, 0.9724 for precision, 0.938 for recall, and 0.9692 for average precision.

The main constraints for the automatic localization of dermoscopic features are related to the very small size of these features; the complexity of their analysis, which requires some expertise; and the lack of annotated images for model training. Based on the results presented in the visual and metrics evaluation, we find that the proposed YDL algorithm can achieve near expert detection despite the constraints associated with this task. Moreover, when comparing the YDL method with two other methods in the literature, the proposed method extracts the most dermoscopic features and achieves better results. These results confirm those obtained with the metrics evaluation.

The applications of the proposed method are multiple. In consultation, it can be used to provide the specialist with a mask of the dermoscopic features present in the image. Knowing that some features are difficult to observe with the naked eye. It can also be used for the automatic classification of skin lesion images. For example, when the globules are atypical (differences in size, shape, color), the diagnosis of melanoma is favored. This principle can be transformed into a rule that is applied to the mask that our method provides to distinguish melanoma from non-melanoma. Finally, our method has the great advantage of generating the annotation file based on predictions, knowing that the annotation of images is a tedious task. This label can be used to annotate unannotated images and, thus, increase the number of annotated images.

Automatic image classification is another approach to improving the accuracy of skin lesion diagnosis. In fact, this is one of the approaches most frequently proposed in the literature [39,41,42,43,44]. Classification involves determining the class to which the lesion belongs. The lesion may be, for example, a melanoma, a basal cell carcinoma, or a nevus. Some references focus on binary classification (benign or malignant) [39] or multi-class classification [41,42]. Kamrul et al. in [41] have proposed a multiclass classification method for dermoscopic images called DermoExpert using convolutional networks. Instead of predicting the class of the entire lesion, our proposed YDL method first predicts the class to which certain areas of the lesion belong. The classes are, therefore, the dermoscopic features (pigment network, negative network, globules, milia-like cysts, or streaks). Secondly, the contours of these zones are delineated to distinguish them from zones that do not have the desired features. Thus, unlike lesion classification, feature detection presents specialists with arguments or elements that enable them to confirm or refute their diagnosis.

## 5. Conclusions

In this paper, a dermoscopic localization feature method-based, single-shot object detection algorithm is proposed as a solution to obtain a complete understanding of dermoscopic images and give physicians more relevant information to increase their accuracy. The objective of this work is to propose a more efficient method than those existing in the literature. To achieve this goal, we proposed an algorithm named YDL, defined in four points: (i) to generate annotations in the JSON format for supervised learning of the model; (ii) to base it on the latest version of Yolo; (iii) to pre-train the model for the segmentation of skin lesions; (iv) to train five models for the five dermoscopic features. The results show an improved prediction with YDL compared to other methods.

One limitation of this work is the number of models used (as many models as dermoscopic features). This choice was made because of the very small size of the dermoscopic elements, the complexity of some structures, and the fact that in an image, the same feature can be found in different places. While this choice provides interesting predictions, one prospect would be to use a single model to locate all dermoscopic features. The future work will explore advanced techniques to achieve this goal. We will also study different pre-processing methods adapted to dermoscopic images that can be used to improve the visibility of dermoscopic features and, thus, their automatic localization. Finally, to address the limited number of images, we plan to combine, in the future work, images with clinical metadata to improve the accuracy of the feature localization model.

## Figures and Tables

**Table 1:** Performances of the YDL algorithm.

Metrics	Globules	Milia-Like Cysts	Pigment Network	Negative Network	Streaks	All Features
DSC	0.984	0.979	1	0.947	0.969	0.9758
JSC	0.970	0.960	1	0.900	0.940	0.954
Precision	0.985	0.946	0.995	0.940	0.996	0.9724
Recall	0.950	0.935	0.992	0.871	0.942	0.938
Average precision	0.980	0.976	0.994	0.915	0.981	0.9692

**Table 2:** Comparison of different dermoscopic feature detection methods.

Method	Techniques	Dermoscopic Structures	Dataset	Estimated Complexity
Kawahara J. [19]	CNN and interpolated map	pigment network, negative network, milia-like cysts, streaks	ISIC	**Low**
LFN [18]	CNN	pigment network, negative network, streaks, milia-like cysts	ISIC	Hight
**YDL**	Yolo	globules pigment network, negative network, streaks, milia-like cysts	ISIC	Hight

**Table 3:** Performances of different dermoscopic feature detection methods that use the ISIC challenge dataset.

Method	JSC	DSC	Recall
Kawahara J. [19]	0.895	-	0.534
LFN [18]	0.833	0.710	0.693
**YDL**	**0.954**	0.976	**0.938**

## Data Availability

Publicly available datasets were analyzed in this study. These data can be found here: https://challenge2018.isic-archive.com (accessed on 4 March 2023).
